# Is Boric Acid Good for Babies?

**Published:** 1915-11

**Authors:** Raymonde A. Albray

**Affiliations:** Newark, N. J.


### IS BORIC ACID GOOD FOR BABIES?

BY RAYMONDE A. ALBRAY, D.D.S., NEWARK, N. J.

In preparing myself to give a series of talks on the teeth to nurses in hospital training schools, I referred to the books which are used by them as text books to learn what they had to say upon the subject. In my reading I found the same method given for the care of the mouth of the baby during the first years of its life in practically every one, and have since seen it in various other books, viz.: "In caring for the mouth of the nursing child, wash it well after each feeding with a three or four per cent. solution of boric acid applied by wrapping a piece of linen or cotton around the finger." After thinking this over for a while I conducted a series of tests and did a little figuring, then I obtained the U. S. Government report on "Influence of Food Preservatives and Artificial Colors on Digestion and Health, vol. 1, Boric Acid and Borax, by H. W. Wiley, M. D., and others." The information obtained from this report confirmed my belief that it is bad practice to use boric acid in a baby's mouth.

All other considerations aside, what reason is there for using the boric acid solution in an infant's mouth? There are no teeth with interproximal spaces to hold food particles, and unless the child have a stomatitis there is no need for an antiseptic wash. The reason for washing a child's mouth after nursing is to remove the particles of milk which may remain in the folds of the mucous membrane, and there is no good reason why this can not be accomplished just as well with plain water or with a normal salt solution, and in the light of the data I will give, would it not be a better method of procedure?

Do not construe the above to mean that the boric acid solution may not be used with good results in apthae or stomatitis, for in these diseased conditions it is sometimes of value. I am questioning the use of the drug in the mouths of infants with healthy oral tissues.

A 3 per cent. solution contains 1.71 grains of boric acid to the drachm, or nearly .3 grain per minim; a 4 per cent. solution contains 2.29 grains per drachm, or nearly .4 grain per minim.

With a piece of gauze or linen, three inches square, wrapped around the finger and dipped into a glass containing the above solutions, it was found that with gentle pressure, about that which would be employed in a child's mouth, from four to ten drops of the solution were dislodged. We all know that the tendency of the nursing child is to suck anything which is put into its mouth, so it is entirely probable that a greater quantity than this is removed from the gauze by the child, particularly as said gauze may be dipped into the solution a number of times during the cleaning process. Probably not all of the solution so removed reaches the child's stomach, some of it may be spit out, but it is certain that some of it does get there. Let us see what this means. But first let me quote from the government report, relative to the action of boric acid when taken into the system by normal men.

"The most interesting of the observations which were made during the progress of the experiments was in the study of the direct effect of boric acid and borax, when administered in food, upon the health and digestion. When boric acid, or its equivalent in borax, is taken into the food in small quantities, not exceeding half a gram (7½ grains) a day, no notable effects are immediately produced. The medical symptoms of the cases, in long-continued exhibitions of small doses or in large doses extending over a shorter period, show in many instances a manifest tendency to diminish the appetite and to produce a feeling of fullness and uneasiness in the stomach, which in some cases results in nausea, with a very general tendency to produce a sense of fullness in the head, which is often manifested as a dull and persistent headache. In addition to the uneasiness produced in the region of the stomach there appear in some instances sharp and well-located pains, which, however, are not persistent.''

''The administration of boric acid to the amount of 4 or 5 grams per day continued for some time results in most cases in loss of appetite and inability to perform work of any kind. In many cases the person becomes ill and unfit for duty.''

''The administration of boric acid to the extent of one-half gram per day yielded markedly different results from those obtained with the larger quantities of the preservatives. This experiment, Series V, conducted as it was for a period of fifty days, was a rather severe test, and it appeared that in some instances a somewhat unfavorable result attended it. On the whole, the results show that one-half gram per day is too much for the normal man to receive regularly.''

''It appears, therefore, that both boric acid and borax, when continuously administered in small doses for a long period or when given in large quantities for a short period, create disturbances of appetite, of digestion and of health.''

We will now return to our argument, and to be entirely on the conservative side, will use the lowest figures obtained, viz., that four minims of a 3 per cent. solution was extracted from the gauze or cotton at each cleaning of the mouth, this process being done after one-half of the feedings (although the writers all say to do it after each nursing), or say five times per day.

I have shown that a minim of a 3 per cent. solution contains nearly three-tenths of a grain, and four minims would therefore contain slightly more than a full grain of boric acid. Thus it is very easy for an infant whose mouth is washed five times a day with a 3 per cent. solution of boric acid to receive a considerable sized dose of the drug in the course of a day, and it reaches the stomach at about the same time the food does, or at least during the process of digestion. If the child swallowed only two minims five times a day, it would have taken two and one-half grains of the drug into its stomach. Dr. Wiley has shown that seven and one-half grains (½ gram) per day is too much for a normal man to receive regularly. In medical practice an infant of one year would be given 1-13 the dose of a drug given an adult; but in using a 3 per cent. boric acid solution to clean the mouth, a child much younger than one year, if it takes 2½ grains into its stomach, as I have shown above, is being given a dose only one-third that which has been shown to be harmful to normal adults, or, in other words, is receiving in proportion considerable more than has rendered a normal man ill and unfit for duty.

Is it not possible that many of the upset stomachs after feeding, which necessitate changing from mothers' milk, because "it does not agree with baby," or indicate a change in formula of the milk or food used in artificial feeding, or that the loss of appetite, fullness in the stomach, colic and nausea may result from the boric acid used in cleaning the mouth of the child? And if the above is possible, and I fully believe that it is, may not permanent injury be done to every baby whose mouth is cleaned with boric acid? It is a common practice among nurses to keep rubber nipples in a boric acid solution, and unless the nipples are thoroughly washed before giving them to the child, may we not be giving them milk containing a quantity of this dangerous preservative and do so unwittingly and with the idea that it is the sterile thing to do?

Thinking people demand food free from preservatives because the government has shown that they are detrimental to health; boric acid is one of these injurious substances, so why run any risk of injuring the health of our children by using it when its use is entirely unnecessary?—*New Jersey Dental Journal.*

